# Death of Prof. Horner

**Published:** 1853-05

**Authors:** 


*Death of Professor* HORNER.

WE copy the following sketch of our old preceptor, Dr. W. E. HORNER, from the *Medical Examiner*, April 9.

DR. WILLIAM E. HORNER, Professor of Anatomy in the University of Pennsylvania, died at his residence in this city on the 13th of March last, in the 60th year of his age. His death, though somewhat sudden, was anticipated by his friends and family for some weeks; and, indeed, for many years, we believe, it had been apparent that organic disease was making slow but sure inroads on his naturally vigorous constitution.

No death, in the profession of this country, could have excited a more general sensation than that of Dr. Horner. Connected for thirty-three years with the University of Pennsylvania, as the adjunct and successor of Physick, he stood in the foremost rank among our teachers of national reputation; while he was no less widely known as the author of Treatise on Special Anatomy and Histology, as a constant contributor to our periodical medical literature, and as a skilful, original, and successful surgeon.

Dr. Horner was born on the 31st of June, 1793, at Warrenton, in Fauquier county, Virginia, where he received his early education. He afterwards spent some time in an Academy at Dumfries, in Prince William County, in the same State, where he remained til his seventeenth year.

In 1810, he commenced the study of medicine with Dr. John Spence, of Dumfries, a Scotch Physician and graduate of Edinburgh, who enjoyed considerable reputation as a practitioner.

In the autumn of 1812, he attended his first course of lectures in the University of Pennsylvania, and soon evinced his predilection for the study of Anatomy, being occasionally employed in assisting the Demonstrator of Anatomy in the preparation of Prof. Wistar's lectures.

On the 3d of July, 1813, he obtained a commission as Surgeon's Mate in the Army of the United States; and was appointed to the regiment stationed at Fort Mifflin, near Philadelphia.

In the Spring of 1814, he graduated at the University, having presented an Inaugural Essay on Gunshot Wounds. He was ordered in the summer of this year, to the Niagara frontier, and was actively employed at Buffalo, Niagara and Fort Erie. Our readers are familiar with his spirited and instructive reminiscences of this campaign, published in late numbers of the Examiner,

After the peace, in 1815, he was stationed at Norfolk, but he soon after resigned his commission, and returned to his native town, Warrenton, Virginia, where he entered upon the practice of his profession. It was not long, however, before he decided to seek a wider sphere, and, in November of the same year he settled permanently in Philadelphia.

Dr. Horner's advancement here was almost without precedent in rapidity. A stranger, without interest or influence, within two years from his establishment in Philadelphia, he entered the University as Demonstrator of Anatomy, under the most distinguished of American teachers; and three years afterwards, (in 1820,) he was appointed adjunct Professor of the same branch. In 1831, on the resignation of Dr. Physick, he was elected Professor of Anatomy in the University, and discharged the duties of this chair till within a short period of his death.

From the date of his appointment to the adjunct Professorship, he devoted himself to the formation of an anatomical cabinet, which has gradually become one of the most splendid and complete in the world.

As a lecturer on Anatomy, Dr. Horner was distinguished for clearness, perspicuity, and simplicity of style, a thorough familiarity with his subject, which secured entirely the confidence and attention of his hearers, and a manly, unaffected delivery. As a *teacher* he had no superior, (as we can bear grateful testimony.) To oratorical talent he made no pretensions, and had no claims.

Though up to a short period of his death, Dr. Horner continued in the steady discharge of his professional and other duties, he had for years suffered from dyspnœa, palpitation, and other symptoms which left little doubt that he labored under cardiac disorder; and the emaciation of his frame made it evident that it was producing serious derangement of the functions of nutrition. For some weeks preceding his death, dropsical effusion had appeared. And, though within a day or two of his death, he was able to participate in the examination of students for degrees, yet we believe that neither himself nor his family entertained any hope that his life could be long protracted. The immediate termination, was, however, somewhat unexpected.

The *post-mortem* examination confirmed the diagnosis of disease of the heart. It was found very considerably hypertrophied and enlarged, being five and a half inches from the apex to the origin of the pulmonary artery, five and three-quarter inches in diameter, and thirteen and a half inches in circumference at its base. The tricuspid and mitral valves were healthy. The arch of the aorta was dilated and thickly ossified. There was also recent peritonitis, with streaks of fresh coagulable lymph over the peritoneal surface of the intestines. Perforation of the stomach or bowels has been suspected, as the cause of the peritonitis, but no traces of this lesion were found.

Dr. Horner's death, at this comparatively early age, is a loss which the profession of our country feel severely. As an anatomist and surgeon, the worthy successor of an illustrious predecessor, his place must long be vacant. But if not full of years, he went full of honors, leaving an unstained reputation in every personal and professional relation. It is gratifying too, to know, that his labors were not without that rare professional recompense—an ample fortune, which he owed exclusively to his own exertions. No man could have closed life under more consolations; and that greatest and best of consolations, a firm Christian hope, had been long and well secured.

